# Protective role of ellagic acid in the adhesive interface after dental bleaching

**DOI:** 10.1038/s41598-025-26885-2

**Published:** 2025-11-07

**Authors:** Lucia Trazzi Prieto, João Victor Frazão Câmara, Josué Junior Araujo Pierote, Lethycia Almeida Santos, Carolina Ruis Ferrari, Débora Alves Nunes Leite Lima, Luís Alexandre Maffei Sartini Paulillo

**Affiliations:** 1 Department of Restorative Dentistry, Piracicaba Dental School, University of Campinas State University of Campinas, Campinas, Brazil; 2 Saarland University, Homburg/Saar, Germany; 3 Department of Biological Sciences, Bauru School of Dentistry, University of São Paulo, Bauru, Brazil

**Keywords:** Polyphenols, Dental bonding, Bond strength, Nanofiltration, Health care, Medical research, Materials science

## Abstract

To evaluate the effect of ellagic acid (EA, extracted from pomegranate) on the quality and durability of the adhesive interface after tooth whitening. Tooth fragments were divided into (*n* = 10/group): G1 - control (no tooth whitening and no restoration), G2 – immediate whitening and restoration and G3 – whitening followed by EA application and restoration. The samples were subjected to an in-office bleaching technique and, the amount of residual oxygen released was measured. Resin composite blocks and sticks were fabricated and bonded to the dental surfaces. After 24 h and 12 months, microtensile bond strength was evaluated by microtensile shear test. The samples were coated with carbon, and the adhesive interface examined by scanning electron microscope (SEM). Data were analyzed with two-way ANOVA followed by Tukey’s test (α = 0.05). According to the bond strength test, higher values were observed in G1 > G2 < G3 at 24 h and 12 months, without statistically significant difference (*p* > 0.05). G1 showed the lowest level of nanoleakage, while G2 exhibited the highest. The prior application of EA in G3 resulted in intermediate nanoleakage. SEM analysis revealed a more stable adhesive interface in G3, with a lower amount of silver nitrate deposition compared to G2. EA appears to have a synergistic effect, qualitatively improving the in vitro quality of the adhesive interface by reducing oxygen in the dental structure. However, further studies are required to elucidate the mechanism of action of EA.

## Introduction

Tooth whitening is a dental procedure that enhances the aesthetics of the smile without requiring invasive interventions such as crowns or veneers^1–3^. However, immediately after bleaching, residual oxygen and other by-products from the bleaching agents remain within the dentinal tubules, interfering with the adhesion process. This condition can persist for up to 2 weeks after the end of bleaching treatment^4,5^. Consequently, the presence of residual oxygen affects the formation of the hybrid layer between the adhesive system and the dental substrate^6,7^. Oxygen retained on the enamel or dentin surface interacts with resin monomers, preventing the formation of a homogeneous hybrid layer by inhibiting or reducing the polymerization reaction^8,9^, which ultimately compromises the bond strength at the tooth-restoration interface.

To address this issue, pretreating dentin with antioxidants has been shown to reduce residual oxygen and improve bond strength values following bleaching. Therefore, the use of agents with antioxidant properties may contribute to the increased clinical longevity of adhesive restorations^10,11^. A 14-day waiting period between bleaching and restorative procedures has been recommended to allow sufficient time for the elimination of residual oxygen and its by-products^12,13^. However, some studies have shown no significant differences in bond strength or resin tag formation when restoration is performed 7, 14 or 21 days after the completion of tooth whitening^14,15^.

Thus, to enhance the elimination of residual oxygen and better define the appropriate timing for adhesive restoration, some researchers have investigated the use of antioxidant enzymes^16,17^. Among these, ellagic acid (EA) is a polyphenol synthesized by various plant species, known for its protective role against ultraviolet light, viruses, bacteria, and parasites. Additionally, ellagic acid is a biphenol classified as a hydrolysable tannins, and it has demonstrated satisfactory in vitro antioxidant potential^18^.

EA is a dimeric derivative of gallic acid, which is widely found in nuts and fruits such as blueberries, strawberries, and pomegranates^19^. It has been reported to possess several biological properties, including antioxidant and anti-inflammatory activities^20,21^. Its antioxidant effect is attributed to its ability to bind metal ions and neutralize hydroxyl, nitrogen, and peroxyl radicals^18^. Furthermore, studies have shown that ellagic acid can inhibit the growth of cancer cells, reduce inflammation, and protect brain function^22,23^. It also exhibits antioxidant potential through metal-chelating and radical-scavenging activities, as well as by modulating the activity of antioxidant enzymes^24^.

In this context, studies evaluating the antioxidant activity of EA on the adhesive interface are still scarce. Therefore, the aim of this study was to in vitro assess the effect of EA on the quality and durability of the adhesive interface following tooth whitening. The null hypothesis tested was that EA does not protect the adhesive interface after tooth whitening.

## Materials and methods

### Ethical aspects

This research was approved by the Human Research Ethics Committee of the University of Campinas (UNICAMP), and the teeth were obtained throught donation, in accordance with resolution No. 466/2012 of the National Health Council and the Declaration of Helsinki. Informed consent was obtained from all participants.

### Tooth sample preparation and experimental groups

Freshly extracted human molars were collected. The teeth were cleaned using Gracey-type periodontal curettes (types 5, 6, 7 and 8; Hu-Friedy, IL, USA), pumice paste (SSWhite, Rio de Janeiro, RJ, Brazil), and water. Then, stored for one week at 4 °C in a 0.5% thymol solution until use^25^.

**Fig. 1:**
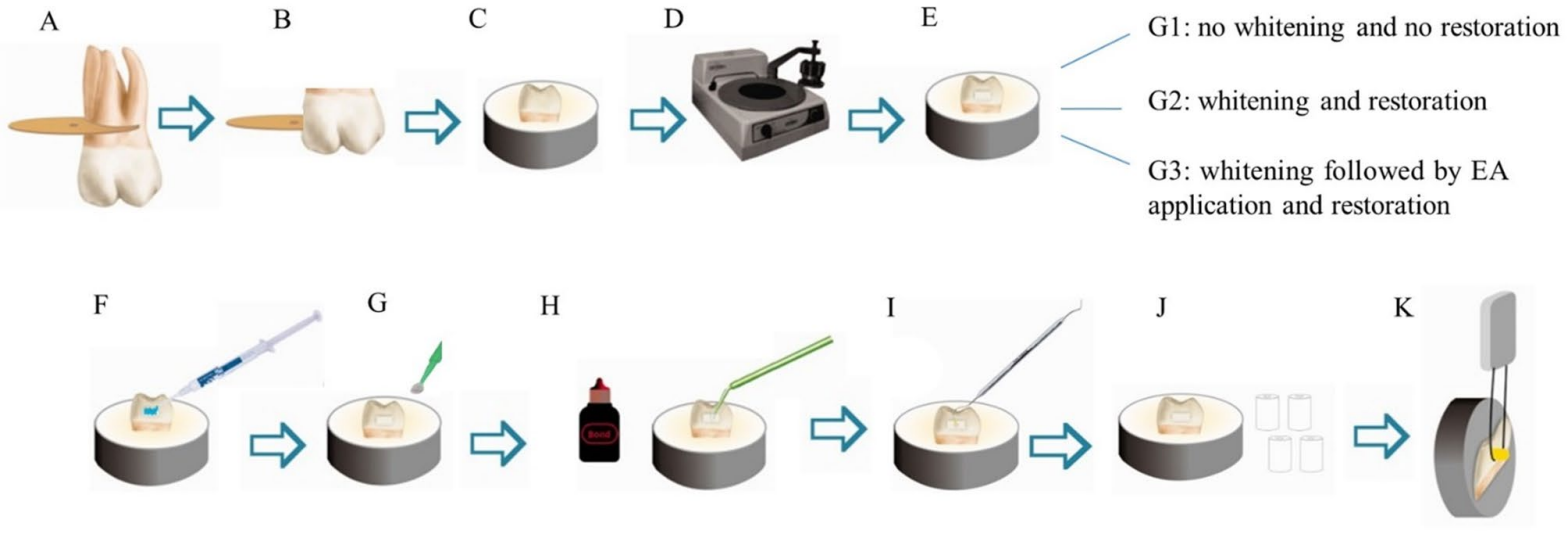
Experimental design of the study. (**A**,**B**) Separation of crown and root, followed by sectioning of tooth fragments to obtain buccal and lingual surfaces. (**C**) Embedding in acrylic resin discs. (**D**) Sample polishing. (**E**) Experimental groups. (**F**) Application of 35% phosphoric acid gel. (**G**) Application of ellagic acid. (**H**) Application of the adhesive system. (**I**) Application of the resin composite. (**J**,**K**) The resin composite block bonded to each fragment was sectioned into multiple sticks for the bond strength test.

A cut was made 3 mm below the amelocementary junction using a precision metallographic cutter (IsoMet 1000, Buehler Inc., Lake Bluff, IL, USA) to remove the roots. Tooth fragments were obtained from the buccal and lingual surfaces of each crown, with an area of 25 mm^2^ (5 mm x 5 mm). A stereoscopic loupe (Leica Microsystems, Wetzlar, Germany) was used to examine the specimens for cracks, fissures or discolorations that could interfere with the whitening procedure. Thirty fragments were selected and randomly assigned to 3 experimental groups (*n* = 10/group): G1 - control (no tooth whitening), G2 – immediate whitening and restoration and G3 – whitening followed by ellagic acid application and restoration. An overview of the experimental design is presented in Fig. 1.

### Whitening procedure

Opalescence Boost 38% hydrogen peroxide (Ultradents Products, INC, South Jordan, UT) was used according to the manufacturer’s instructions. The gel was applied to the tooth fragment for 15 min, then removed with a suction cannula and rinsed off with water. This procedure was repeated 3 consecutive times per clinical session. A total of 3 clinical whitening sessions were performed, with a one-week interval between each session.

### Ellagic acid

Ellagic acid (EA) was purchased from Sigma Chemical Co. (St. Louis, MO, USA). A 10 µmol/L of EA (in 0.1% DMSO) was prepared based on previous study^16^. The solution was applied to the surface of the fragment using a microbrush in a circular motion for 60 s. Following this, the samples were rinsed with a water spray for 30 s and then stored in jars containing deionized water.

### Adhesive restorative procedure

The procedure was performed according to the manufacturer’s instructions. Conditioning was carried out using 35% phosphoric acid gel (ScotchbondTM, 3 M ESPE, St. Paul, MN, USA) for 15 s, followed by rinsing with water for 30 s. The dental fragment was then gently dried with absorbent paper to preserve dentin moisture. The conditioned dental fragment was dried using absorbent paper to maintain dentin moisture^5^. After the application of ellagic acid, the adhesive system (Adper Single Bond 2, 3 M ESPE) was applied and light-cured for 10 s using the RadiiCal high-power LED device (SDI, São Paulo, SP, Brazil).

### Titrations

The first titrimetric analysis was performed immediately after the bleaching treatment, with the value from the control group considered the baseline. Following the application of ellagic acid, the fragment was stored for either 24 h or 12 months, after which a new analysis was conducted. Titrimetric analyses of dissolved oxygen, modified by azide iodide, were carried out using a graduated pipette with the tip immersed in the deionized water in which the specimen had been stored. To each sample, 1mL of 50% manganese solution and 1mL of alkaline azide iodide solution were added, followed by vigorous shaking. A brown precipitate of manganese hydroxide formed and was allowed to settle for 15 min. Subsequently, 2 ml of 85% phosphoric acid was added, and the contents were mixed thoroughly. The precipitate dissolved, releasing free iodine into the solution, initiating the iodine titration with a standard sodium thiosulfate solution. When the solution turned pale yellow, 2 ml of starch indicator was added, and titration continued until the solution became colorless. The dissolved oxygen concentration was calculated in mg/L^-1^; 1 ml of thiosulphate = 1 mg of dissolved oxygen^26^.

### Specimens preparation for the bond strength test

After the adhesive procedure, a 5 mm x 5 mm composite resin block was fabricated using an addition silicone guide with FiltekTM Z250 composite (3 M ESPE). The resin was placed in three increments of approximately 2 mm each, totaling a height of 6 mm. Each increment was light-cured for 20 s using the RadiiCal high-power LED device (SDI, São Paulo, SP, Brazil). 24 h after the restorative procedure, the blocks were sectioned longitudinally along the long axis of the fragment using a precision metallographic cutter (IsoMet 1000, Buehler Inc., Lake Bluff, IL, USA), producing slices approximately 0.8 mm thick. The fragment was then rotated 90°, and additional cuts were made to obtain sticks with an adhesive area of approximately 0.64 mm^2^. The number of slices that fractured prematurely during handling was recorded. Half of the sticks obtained for the microtensile test were analyzed after 24 h, while the remaining half were stored in distilled water—replaced weekly—for testing after 12 months. All samples were stored at a temperature of − 20 °C^25^.

### Bond strength test

For this assay, the composite resin block bonded to each fragment was sectioned into multiple sticks for the bond strength test used. The thickness of the cross-section at the adhesive interface was measured with a digital caliper (Mitutoyo Sul Americana, Suzano, SP, Brazil), and the adhesive area (in mm^2^) was recorded to calculate the bond strength values in Megapascals (MPa). The sticks were mounted on a EZ-Test L universal testing machine (Shimadzu co, Kyoto, Japan) and secured at both ends using a cyanoacrylate-based adhesive (Super Bonder Gel, Loctite, Itapevi, SP, Brazil). The adhesive interface was positioned perpendicular to the direction of the applied force, and tensile testing was performed at a crosshead speed of 0.5 mm/min using a 20 kg force (kgf) load cell, until specimen failure occurred. At the point of rupture, the machine automatically stopped, and the microtensile bond strength values were tabulated (kgf). The bond strength (MPa) was calculated using the formula: Bond strength (MPa) = (Fracture load in kgf × 9.8) / Adhesive area (mm^2^). All values were coverted to MPa, tabulated and submitted to statistical analysis. The sticks were stored in distilled water to avoid direct exposure to the storage medium and kept at a temperature of -20 °C^25^.

### Nanoinfiltration and scanning electron microscope (SEM)

Three slices from each group (24 h and 12 months) were immersed in a 50% ammoniacal silver nitrate solution. The solution was prepared by dissolving 10 g of silver nitrate crystals in 10 mL of deionized water, by the addition of drops of 28% ammonium hydroxide until the solution cleared. The samples were submerged in this solution for 24 h at 37 °C, then rinsed in distilled water for 2 min until completely clean. Subsequently, they were immersed in a developer solution for 8 h and exposed to direct fluorescent lighting (via a lamp) and indirect fluorescent lighting (via ambient lighting)^26^.

The samples were processed for scanning electron microscope (SEM) to visualize nanometric spaces within the hybrid layer. They were embedded in polystyrene resin and sequentially ground using 600, 1200, and 2000 grit sandpaper on a metallographic polishing machine. After grinding, polishing was performed using felt disks and diamond pastes of decreasing grits (3 μm, 1 μm and 0.25 μm). Between each grit change, the samples were immersed in distilled water in an ultrasonic tank for 10 min to remove surface debris. Next, the samples were dried with absorbent paper and etched in 85% phosphoric acid for 30 s, rinsed in distilled water, and immersed in a 10% sodium hypochlorite solution for 10 min. After being washed (2x) and dried at room temperature, the samples were dehydrated in a graded ethanol series (25%, 50%, 75%, 90%, and 100%), 10 min per step^26^. Finally, the samples were coated with carbon for observation under SEM (JSM 5600 LV, Jeol, Tokyo, Japan) at 50x magnification, operating under high vacuum at 20 kV. Backscattered electron images were obtained, and the silver nitrate-infiltrated areas were calculated using Image Processing and Analysis in Java (ImageJ, version 1.53t, National Institutes of Health, USA. https://imagej.net/ij/) software for qualitative analysis^26^.

### Statistical analysis

Statistical analysis was performed to determine which technique was most effective. The data were analyzed using two-way ANOVA with the SAS statistical software (version 9.1.3; SAS Institute Inc., Cary, NC, USA), followed by Tukey’s post hoc test. A significance level of α = 0.05 was adopted.

## Results

### Bond strength test

**Fig. 2:**
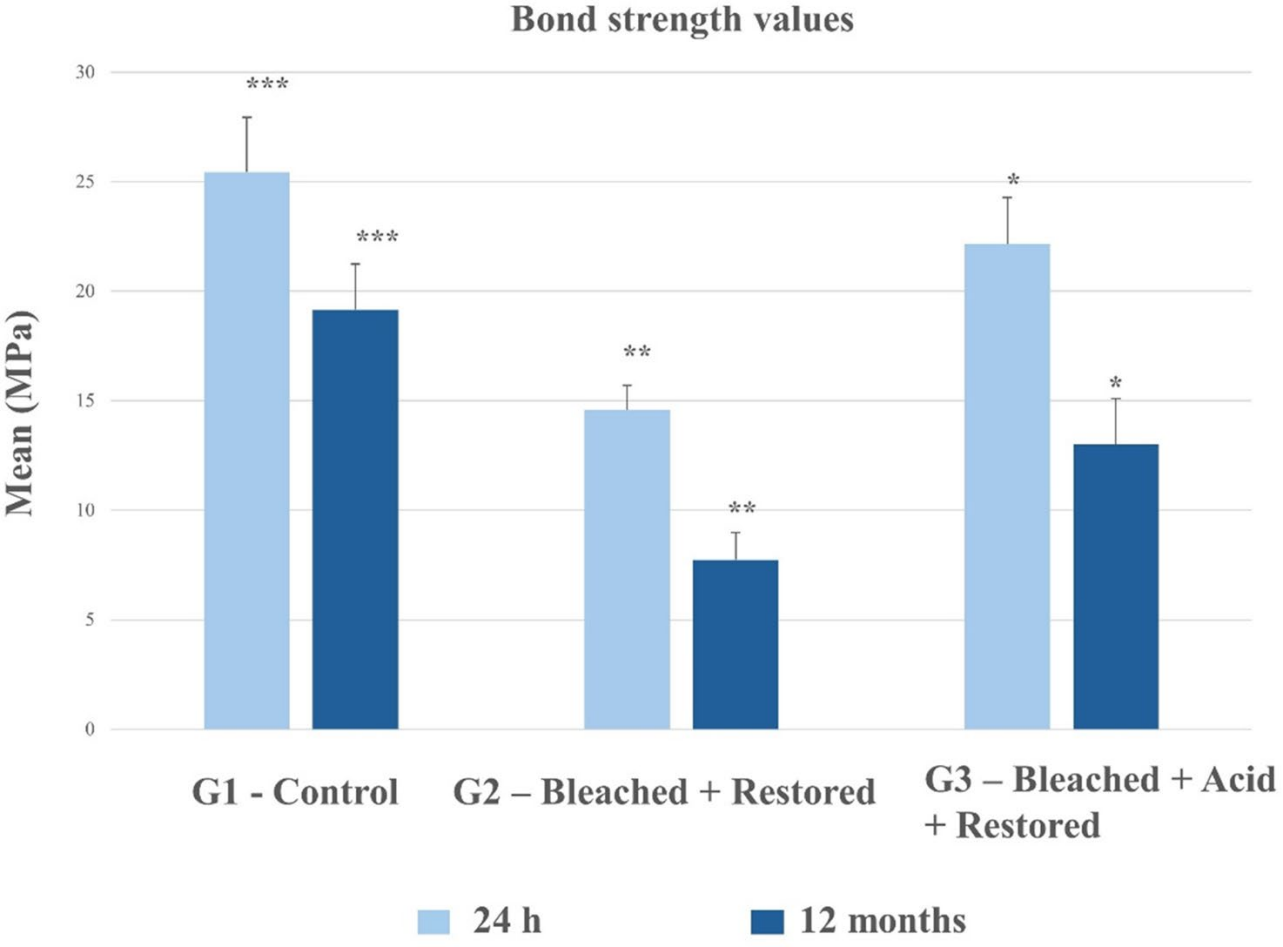
Bond strength values (MPa) of the groups after 24 h and 12 months of storage (*n* = 10/group, Mean SD). Averages followed by symbols (*) differ statistically by the Tukey t-test (*p* > 0.05).

For group 1, the bond strength values after 12 months of storage showed no statistically significant difference compared to those observed after 24 h (*p* > 0.05). Group 2 exhibited the lowest bond strength values at both time points when compared to the other groups. In group 3, the bond strength values were not statistically different from the control group at 24 h, however, a reduction was observed after 12 months of aging (Fig. 2).

### Nanoinfiltration and scanning electron microscope

Samples aged for 12 months in deionized water showed a slight increase in nanoleakage values, regardless of the group evaluated. As expected, G1 showed the lowest values, particularly in the 24-hour specimens. On the other hand, G2 presented the highest nanoleakage values, especially after 12 months of storage. G3, which received a prior application of ellagic acid, showed intermediate nanoleakage values when compared to the other groups (Table 1).

**Table 1:** Silver nitrate infiltration percentage values according to storage time (*n* = 10/group, mean ± standard deviation).

Groups	*P* value	Mean ± SD
24 h		
G1- Negative Control	*p* > 0.10	13.31 ± 1.12
G2- Bleached + Restored	*p* = 0.09	25.44 ± 0.79
G3- Bleached + Restored + Acid	*p* > 0.10	23.02 ± 0.66
12 months		
G1- Negative Control	*p* < 0.001	7.74 ± 1.24
G2- Bleached + Restored	*p* < 0.001	19.2 ± 2.08
G3- Bleached + Restored + Acid	*p* < 0.001	13.2 ± 2.08

**Fig. 3:**
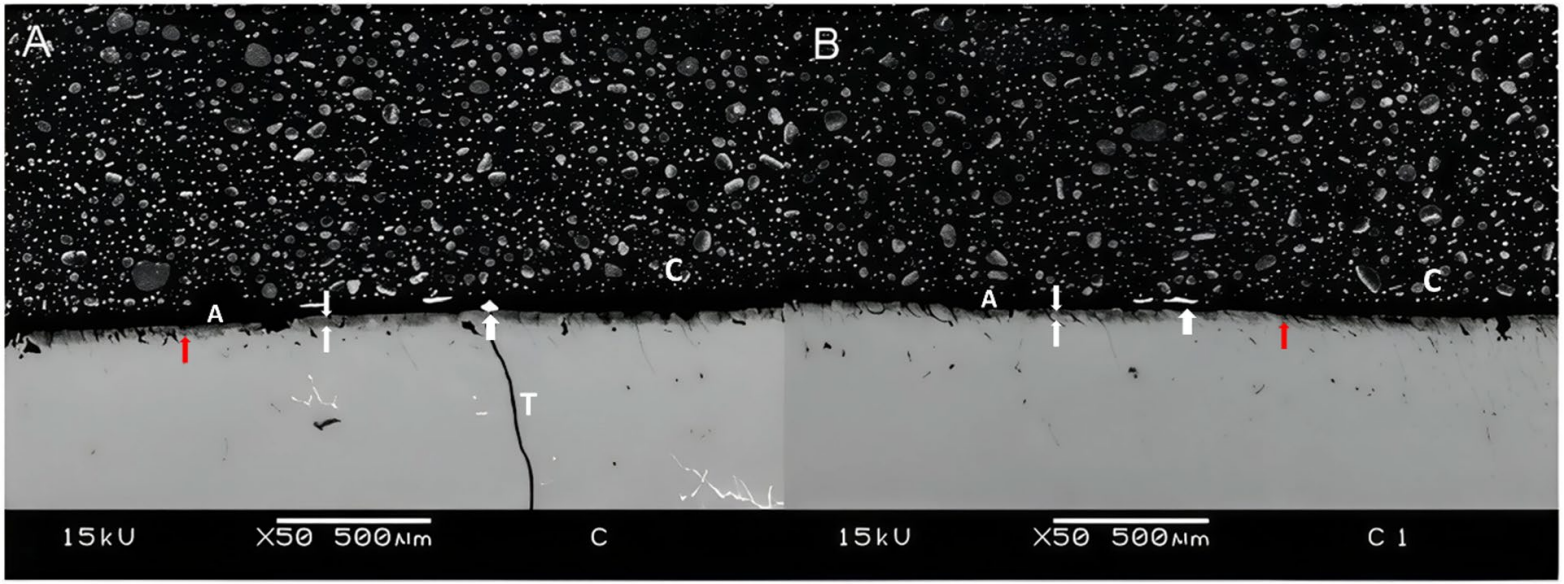
Representative image of interfacial silver nanoleakage from SEM (50×, scale bar = 500 μm) in the control group after 24 h (**A**) or 12 months (**B**). Minimal deposition of silver nitrate is observed in A, while a similar pattern of nanoleakage is observed after 12 months of storage in B. The white arrow indicates ammoniacal silver nitrate; red arrows indicate nanoleakage; T resin tag; the area between the two white arrows represents the hybrid layer; C composite; A adhesive.

**Fig. 4:**
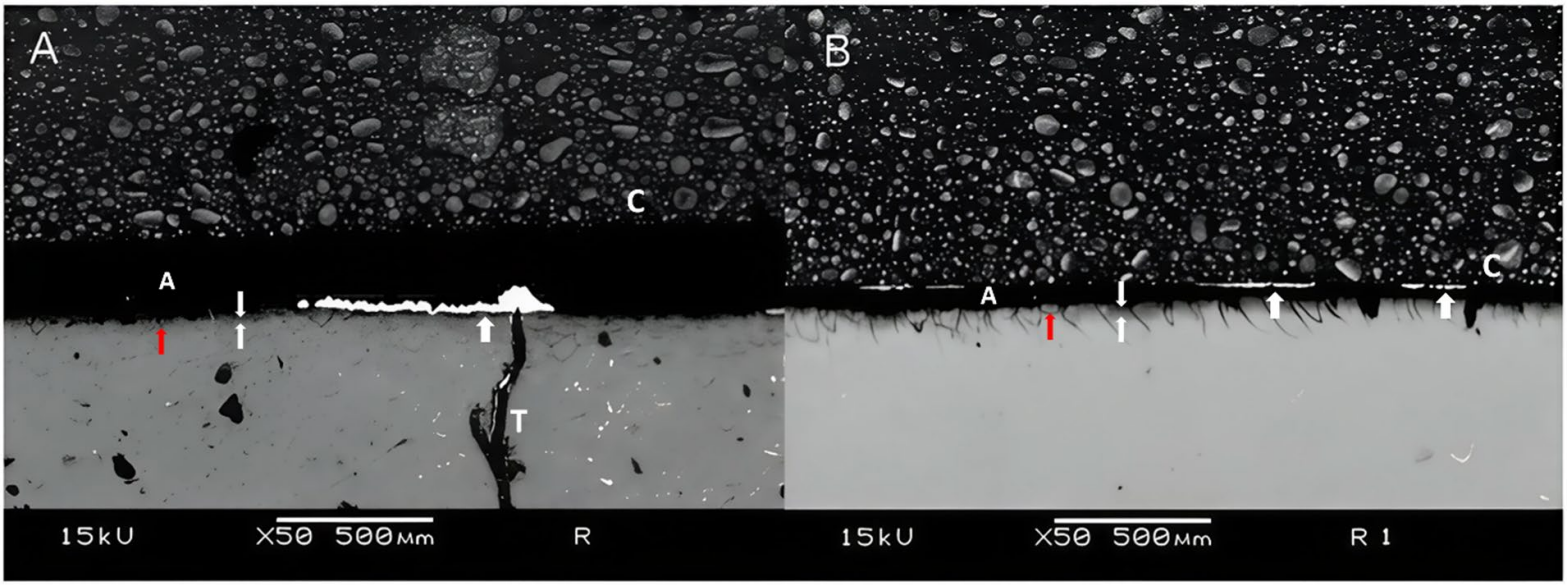
Representative image of interfacial silver nanoleakage from SEM (50×, scale bar = 500 μm) in the group 2 after 24 h (**A**) or 12 months (**B**). Extensive nanoinfiltration was observed, both 24 h and 12 months after storage, as seen in A and B. The white arrow indicates ammoniacal silver nitrate; red arrows indicate nanoleakage; T resin tag; the area between the two white arrows represents the hybrid layer; C composite; A adhesive.

**Fig. 5:**
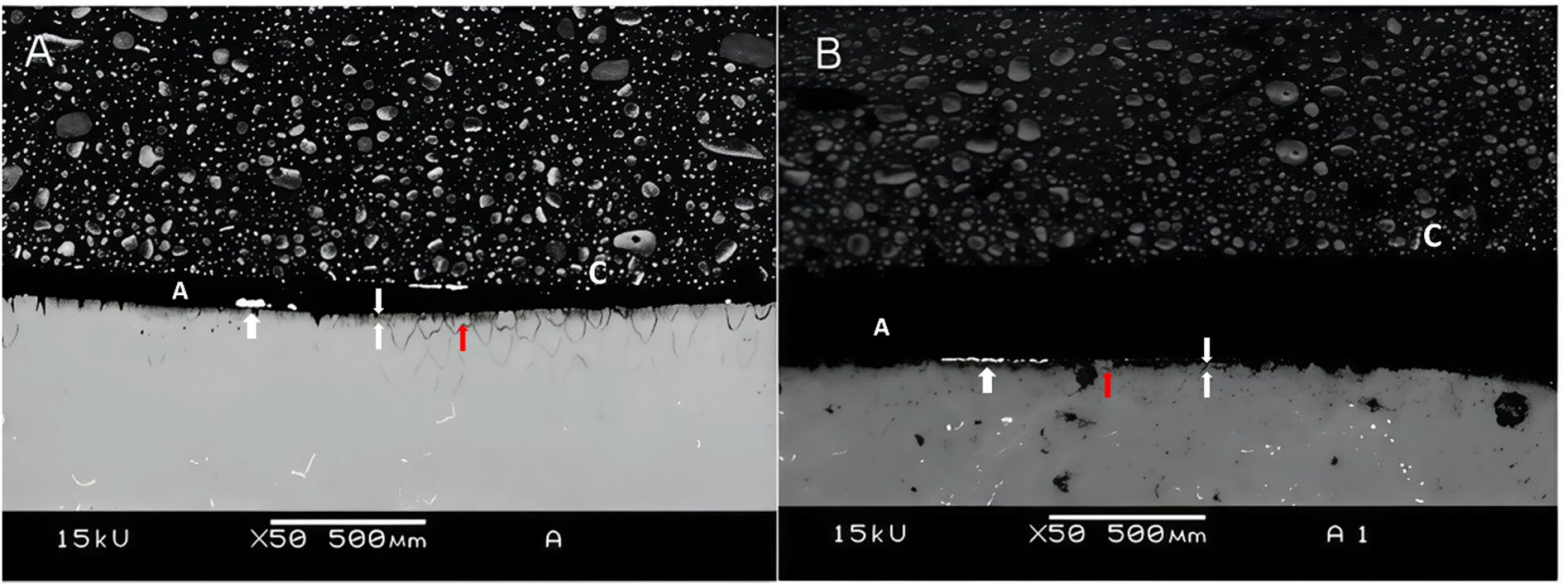
Representative image of interfacial silver nanoleakage from SEM (50×, scale bar = 500 μm) in the group 3 after 24 h (**A**) or 12 months (**B**). A smaller amount of nanoinfiltration was observed compared to the other groups, with a slight increase in nanoinfiltration after 12 months compared to 24 h. The white arrow indicates ammoniacal silver nitrate; red arrows indicate nanoleakage; the area between the two white arrows represents the hybrid layer; C composite; A adhesive.

Figures 3, 4 and 5 are representative SEM images of each group. According to the qualitative evaluation of the samples, a slight deposition of silver nitrate was observed (Fig. 3A), along with similar nanoleakage patterns after 12 months of storage (Fig. 3B). Extensive nanoleakage was noted in G2 at both 24 h and 12 months (Fig. 4A,B). In G3, which contained ellagic acid, a lower level of nanoleakage was observed in comparison to the other groups (Fig. 4), although a slight increase was seen after 12 months compared to the 24-hour specimens. Notably, in G3, improved sealing was evident, with longer resin tags and a thicker, more homogenous hybrid layer. As expected, extensive silver precipitates were observed along the adhesive interface during the immediate evaluation of G2 (Fig. 3; Table 1), whereas significantly fewer precipitates were observed in the ellagic acid group (Fig. 5; Table 1).

## Discussion

The present study compared three adhesive interfaces: control (Group 1), dental bleaching with restoration (Group 2), and dental bleaching with ellagic acid (EA) application prior to restoration (Group 3) for nanoleakage and nanoinfiltration. The null hypothesis is partially rejected, as a lower level of nanoleakage was qualitatively observed after ellagic acid application. However, no specimens were free of microleakage or microgap formation. This finding is consistent with previous studies that demonstrate gingival margins located below the dentin-enamel junction (DEJ) present a higher risk of marginal leakage in class I resin composite restorations compared to the margins placed above the DEJ^27–30^. The complex nature of the dentin substrate - characterized by a 45% inorganic composition, randomly arranged dentinal hydroxyapatite within an organic matrix primarily composed of type I collagen^31–35^ - and its intimate connection with the pulp through numerous fluid-filled tubules from the pulp to the DEJ^36,37^ complicate bonding. The presence of dentinal fluid can interfere with adhesion, as hydrophobic resins do not effectively bond to hydrophilic substrates, even when resin tags form within the dentin tubules^38^.

Aesthetic anterior restorations often require replacement following bleaching, as they do not respond to whitening agents in the same manner as tooth structure. A 14-day waiting period before restorative procedures is typically recommended to allow the dissipation of residual oxygen and by-products, ensuring optimal bond strength^39–43^. To accelerate this process and better define the necessary interval before adhesive restoration, this study is the first to investigate the use of ellagic acid - due to its antioxidant properties - for reducing residual oxygen post-bleaching and potentially shortening the delay between bleaching and restoration.

Ellagic acid is a natural antioxidant found in various fruits and vegetables, including chestnuts, raspberries, strawberries, blueberries, walnuts, and pomegranates. It has demonstrated properties against breast, pancreas, esophagus, colon, skin and prostate cancers, as well as protective effects against cardiovascular diseases and skin pigmentation disorders. In Dentistry, the application of ellagic acid prior to adhesive procedures may reduce residual oxygen after bleaching, thereby minimizing its interference with the resin polymerization and potentially enhancing bond strength. However, the efficacy of this approach depends on several factors, including the concentration of ellagic acid, application time, and the specific clinical protocol used. While preliminary findings are promising, further research is needed to quantify its effects and to optimize application protocols for clinical use.

In relation to our previous study^44^, a different storage period was adopted, as no statistically significant differences were observed after 6 months. In the present study, the improved performance of the DMSO and ellagic acid combination may be attributed to enhanced wettability of the dentin organic matrix exposed by acid etching^45^, as well as reduced dentinal fluid flow into the hybrid layer during in vitro bonding. These factors may explain the observed differences. It is also possible that DMSO acted as a solvent for the adhesive components, contributing to the protection of the adhesive interface after dental bleaching (G3).

One limitation of this study is the sample size; however, as the first to investigate the interaction between ellagic acid, the dental substrate, and the adhesive interface following tooth bleaching, it serves as a foundation for future research. Further in vitro studies are warranted to validate these findings, given the known systemic and oral health benefits of ellagic acid. In addition, future studies should explore various post-bleaching time intervals (48 h, 72 h, 1 week, 2 weeks, and 1 month) to determine the optimal timing for resin restoration following ellagic acid treatment. Moreover, incorporating ellagic acid into mouthwashes or toothpastes should be explored in future in situ and in vivo studies.

According to our findings, silver uptake was lower in the group treated with ellagic acid compared to Group 2, which may be attributed to enhanced protection of the adhesive interface. Additionally, SEM analysis enabled a qualitative assessment of hybrid layer degradation. Greater nanoleakage and hybrid layer failure were observed in Group 2 than in group Group 3; however, there was no statistically significant difference were found between the groups regarding bond strength values. The complex and sometimes contradictory relationship between bond strength and nanoleakage warrants further investigation. One possible explanation for this discrepancy is the polymerization shrinkage of the restorative material used. Future studies should consider evaluating different resin composites associated with ellagic acid. In the present study, SingleBond 2 - an etch-and-rinse adhesive - was used to assess the effect of ellagic acid on interface bonding durability. In this system, collapsed collagen fibrils can be rehydrated and re-expanded due to the presence of the hydrophilic monomer HEMA, which reduces water evaporation. It is also worth noting that the adhesive used may have interacted negatively with certain molecular chains of ellagic acid, potentially affecting the bonding performance. Therefore, comparing different adhesive systems in future experimental designs is recommended. Moreover, the samples were stored in deionized water after EA application to prevent potential chemical interactions between the teste solution and the various components found in artificial saliva, such as ions, enzymes, or organic molecules. Because EA has known chelating and antioxidant properties, its interaction with calcium, phosphate, or protein components in artificial saliva could have influenced its activity or interfered with the adhesive interface. Deionized water, being chemically inert and standardized, was chosen to minimize confounding variables and ensure better control of the experimental conditions. However, future studies involving in situ or in vivo conditions should consider artificial saliva or more biologically relevant media to simulate the oral environment.

To date, the only polyphenol studied at the adhesive-dentin interface has been epigallocatechin-3-gallate (EGCG), which has demonstrated the ability to preserve dentin bond strength, reduce interfacial nanoleakage after collagenase ageing, suppress MMP activity, and inhibit biofilm formation^46^. Therefore, it is important to highlight that no studies have yet investigated ellagic acid in this context, making data comparison difficult. Nevertheless, the findings presented open new avenues for incorporating this compound into Restorative Dentistry.

## Conclusions

Under the protocol of this in vitro study, qualitative analysis revealed protection of the adhesive interface following the application of ellagic acid. However, further studies are needed to investigate the mechanical, chemical, and biological effects of ellagic acid in association with dental substrates.

## Data Availability

The datasets used and/or analysed during the current study available from the corresponding author on reasonable request.
